# What do we know about growth of vessel elements of secondary xylem in woody plants?

**DOI:** 10.1111/brv.12785

**Published:** 2021-08-09

**Authors:** Adam Miodek, Aldona Gizińska, Wiesław Włoch, Paweł Kojs

**Affiliations:** ^1^ Polish Academy of Sciences Botanical Garden – Centre for Biological Diversity Conservation in Powsin Prawdziwka 2 02‐973 Warsaw Poland; ^2^ Institute of Biology University of Opole Oleska 22 45‐052 Opole Poland

**Keywords:** vessel element, mechanical stress, diurnal strain, vascular cambium, ring‐porous wood, diffuse‐porous wood, intrusive growth

## Abstract

Despite extensive knowledge about vessel element growth and the determination of the axial course of vessels, these processes are still not fully understood. They are usually explained as resulting primarily from hormonal regulation in stems. This review focuses on an increasingly discussed aspect – mechanical conditions in the vascular cambium. Mechanical conditions in cambial tissue are important for the growth of vessel elements, as well as other cambial derivatives. In relation to the type of stress acting on cambial cells (compressive *versus* tensile stress) we: (*i*) discuss the shape of the enlarging vessel elements observed in anatomical sections; (*ii*) present hypotheses regarding the location of intrusive growth of vessel elements and cambial initials; (*iii*) explain the relationship between the growth of vessel elements and fibres; and (*iv*) consider the effect of mechanical stress in determining the course of a vessel. We also highlight the relationship between mechanical stress and transport of the most extensively studied plant hormone – auxin. We conclude that the integration of a biomechanical factor with the commonly acknowledged hormonal regulation could significantly enhance the analysis of the formation of vessel elements as well as entire vessels, which transport water and minerals in numerous plant species.

## I. INTRODUCTION

**Fig 1:**
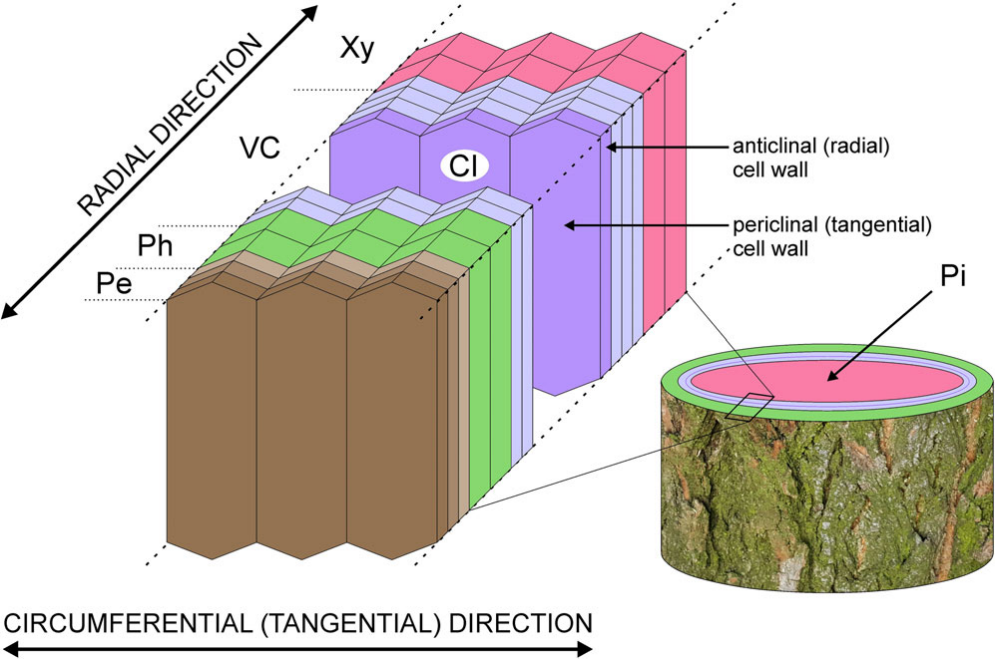
Scheme showing position of vascular cambium and cambial derivatives deposited on the xylem and phloem side within a tree stem. Cambial initials of the vascular cambium are shown in dark violet. Other undifferentiated cambial cells are shown in light violet. Cells of the vascular cambium are purposely spaced apart in order to indicate the periclinal and anticlinal walls of the cambial initials. Pith and periderm are also shown. Periderm (phelloderm, phellogen and phellem) is marked in different hues of brown. Phellogen is responsible for the deposition of phelloderm and phellem. The proportional thickness (number of cells) of different tissues is not taken into account. CI, cambial initials; Pe, periderm; Ph, phloem; Pi, pith; VC, vascular cambium; Xy, xylem.

**Fig 2:**
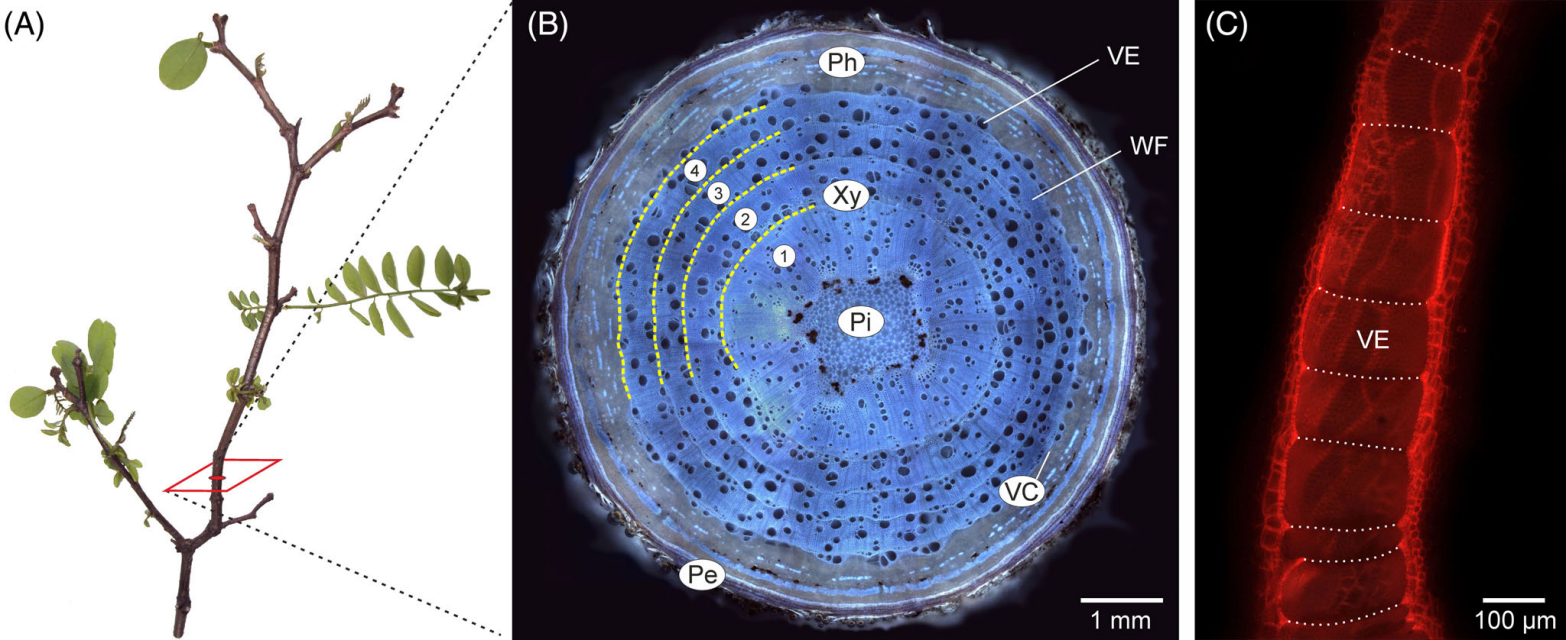
Structure of a branch of *Robinia pseudoacacia* L. (A) Macroscopic view. (B) Whole‐branch transverse section. Yellow dashed lines indicate fragments of growth‐ring boundaries. (C) Tangential longitudinal view of a single vessel comprised of individual vessel elements. White dotted lines show location of perforation plates. Pe, periderm; Ph, phloem; Pi, pith; VC, vascular cambium; VE, vessel element; WF; wood fibre; Xy, xylem. 1, 2, 3, 4, sequential (complete) annual increments.

Processes of growth and differentiation of cambial derivatives, deposited by the vascular cambium – the lateral plant meristem responsible for production of secondary xylem and secondary phloem cells (Crang, Lyons‐Sobaski & Wise, 2018) (Fig. 1) – are extremely complex and insufficiently explained. Even such basic issues as functioning of the vascular cambium and the development of derivative cells, the mechanism of increase in circumference of the cambial cylinder (Włoch *et al*., 2009, 2013; Wilczek *et al*., 2014*a*; Miodek *et al*., 2021), and the course of intrusive growth of cambial initials, vessel elements, and wood fibres (Jura *et al*., 2006; Wilczek *et al*., 2018; Gizińska, Miodek & Kojs, 2021) still remain debated. One of the key processes related to cambial activity is xylogenesis, i.e. wood formation, with its underlying mechanisms of growth of vessel elements and vessel pattern formation. Mature vessel elements are dead cells, devoid of protoplasts, connected to each other by perforation plates, and forming long strands – vessels (Evert, 2006; Fig. 2). These are specialised for conduction of water and minerals from the roots to the leaves. Vessels can take various sizes, both in length and diameter (Zimmermann, 1982). Moreover, dicotyledonous trees show distinct distribution patterns of vessels with different tangential and radial dimensions within their annual growth rings (Beck, 2010) which are classified into different wood porosity types. In ring‐porous wood earlywood vessels are characterized by much larger transverse dimensions than latewood vessels; diffuse‐porous wood vessels have similar diameters throughout the annual growth ring; and semi‐ring porosity represents an intermediate condition (IAWA Committee, 1989). Woody plants with different porosity types adapt differently to various environmental conditions, with an example of such adaptation being how trees respond to the onset of a new growth season (e.g. after winter frosts) (discussed in Gizińska *et al*., 2015). Thus, understanding the processes of growth of vessel elements and pattern formation of water‐conducting system is of great interest (Zasada & Zahner, 1969; Zimmermann, 1983; Zakrzewski, 1991; Kitin, Sano & Funada, 2003; Wilczek *et al*., 2011*b*; Gizińska *et al*., 2021).

There is much information available concerning the hormonal regulation of cambial activity and the formation of vessel elements (e.g. Zakrzewski, 1983; Uggla *et al*., 1996; Uggla, Mellerowicz & Sundberg, 1998; Kramer, 2002, 2006; Schrader *et al*., 2003; Nilsson *et al*., 2008; Petersson *et al*., 2009; Vanneste & Friml, 2009; Grunewald & Friml, 2010; Huang *et al*., 2010; Santos *et al*., 2010; Zhang *et al*., 2010; Aloni, 2013, 2015, 2021; Rutschow, Baskin & Kramer, 2014; Bhalerao & Fischer, 2017). However, in order to understand how vessel elements increase their transverse dimensions (radial and tangential), it is necessary to pinpoint the factors responsible for determining the course of growth of a vessel element, in addition to the commonly accepted contribution of auxin (Aloni, 2007, 2013, 2015; Vanneste & Friml, 2009; Grunewald & Friml, 2010). One such factor is mechanical stress, which seems to play an important role in most, if not all, plant tissues (Hamant *et al*., 2008; Hamant & Traas, 2010; Nakayama *et al*., 2012; Landrein & Hamant, 2013; Nick, 2013; Hamant & Haswell, 2017; Ackermann & Stanislas, 2020; Du & Jiao, 2020; Sampathkumar, 2020; Ramos, Maizel & Alim, 2021; Trinh *et al*., 2021) including cambium and developing xylem (Hejnowicz, 1980, 1997, 2012; Kwiatkowska & Nakielski, 2011; Kojs, 2013). The role of mechanical pressure in maintaining the functionality of the cambial tissue and progression of xylogenesis was first shown by Brown & Sax (1962), who found that longitudinal incisions of tree‐stems, reaching into the xylem led to callus formation due to the removal of mechanical stress. Callus is composed of large cells with thin walls, and is formed not only after injury but also in tissue culture (Beck, 2010). Initial cells of the intact vascular cambium have specific shapes depending on their type. Fusiform initials are axially elongated with small radial dimensions and give rise to the axial system of cells (tracheids, vessel elements, axial parenchyma, and wood fibres). Ray initials have an isodiametric shape and give rise to the radial system of cells (i.e. they are responsible for the formation of rays within the xylem and phloem) (Hejnowicz, 2012; Crang *et al*., 2018). Brown & Sax (1962) showed that the normal pattern of tissue differentiation was restored at incision sites after the formation of new phellogen and cambium. The important role of mechanical stress in the differentiation of xylem cells was confirmed by the lack of radial growth in *in vitro* cultures of cambial cells; cells of the cambium deprived of adjoining layers of xylem and phloem, and thus of their specific mechanical environment, take a spherical shape (discussed in Srivastava, 1973). Lintilhac & Vesecky (1984) found that applying external compressive forces induces spatially ordered divisions in *in vitro* culture of pith tissue.

The influence of mechanical conditions on the growth of vessel elements is extremely interesting, which is reflected in the most recent trends in plant sciences dealing with influence of mechanics on morphogenesis (Robinson *et al*., 2013; Ackermann & Stanislas, 2020; Du & Jiao, 2020; Sampathkumar, 2020; Ramos *et al*., 2021) and ongoing discussion regarding the types of stress occurring in the vascular cambium. There are partially opposing views regarding the type of mechanical stress (compressive *versus* tensile) that acts upon cambial cells in a radial direction (e.g. Hejnowicz, 1997, 2012; Kojs, 2012; Kojs, Malik & Wistuba, 2012). Cambial cells and their derivatives show two basic types of growth – symplastic or intrusive (Wilczek *et al*., 2018; Gizińska *et al*., 2021; Miodek *et al*., 2021). The main type of growth in radial enlargement of a stem is symplastic (Miodek *et al*., 2021). During symplastic growth, the surfaces of the walls of adjacent cells do not move relative to each other, so contacts between cells do not change and cells grow in a coordinated manner (Tulik, 2007). When individual regions of cell walls grow at different rates, this can lead to changes in contacts between cells, and such growth is defined as intrusive growth (Sinnott & Bloch, 1939; Gizińska *et al*., 2021). Intrusive growth plays an important role in the vascular cambium and developing xylem (Jura *et al*., 2006; Wilczek *et al*., 2011*a*,*b*, 2014*a*; Hejnowicz, 2012), such as participating in cambial cell rearrangement which translates into the specific structure and properties of the wood. During development of secondary xylem of angiosperm trees two types of cells show intrusive growth – fibres (Wilczek *et al*., 2018) and vessel elements (Gizińska *et al*., 2021). Intensive intrusive growth of vessel elements can be observed near the border of the cambium and the zone of xylem development. It is therefore possible that the mechanical conditions in which vessel elements and cambial initials grow intrusively are similar. Furthermore, can intrusive growth in both the vascular cambium and the zone of xylem cell development be explained similarly by specific mechanical conditions? To date, anatomical studies have considered the locations and directions of intrusive growth for specific types of cells separately (i.e. cambial initials, wood fibres, and vessel elements). A consequence of this approach may be a failure to understand the higher levels of organization of the complex adaptive system represented by a tree. It may be speculated that in the control of developmental processes, both the biophysical/biomechanical and biochemical/molecular aspects operate in a complementary manner. Below we attempt to elucidate the relationship between the location of intrusive growth of vessel elements and cambial initials, while taking into account the biomechanical background of these processes. Most importantly, this review aims to highlight the potential role of biomechanical forces in vessel element formation. A full explanation of the functioning of the vascular cambium will be possible only by considering all physiological, anatomical, and biomechanical factors involved.

## II. DEFINITIONS

**Fig 3:**
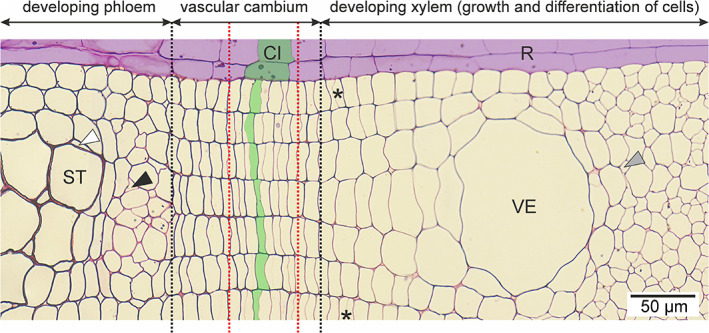
Transverse section of vascular cambium, developing xylem and phloem cells of a *Robinia pseudoacacia* L. stem. Vascular cambium as used in this review includes all undifferentiated cells located between the developing cells of secondary xylem and phloem (area between black dotted lines). The approximate position of the vascular cambium, defined according to the ‘broad defintion’ (Hejnowicz, 2012) as a zone of dividing cells located between secondary xylem and phloem, is marked by red dotted lines. The probable location of cambial initials (fusiform and ray initials) (vascular cambium; narrow definition) is marked in green. The ray is shaded in violet. Asterisks indicate thin cell walls within some recently divided xylem mother cells. White arrowhead, companion cell; black arrowhead, growing tip of phloem fibre; grey arrowhead, growing tip of wood fibre. CI, cambial initial; R, ray; ST, sieve tube; VE, vessel element.

Vascular cambium can be defined in various ways (Hejnowicz, 2012). In the narrow definition the cambium is considered to be a single layer of initials, i.e. meristematic cells that are constantly dividing, periclinally as well as anticlinally (Fig. 3). Their derivatives, which can still divide periclinally (mother cells), are included together with the initials in the ‘cambial zone’ (Wilson, Wodzicki & Zahner, 1966). Application of the narrow definition is rather problematic, as in many cases we are unable to precisely identify cambial initials among periclinally dividing cells. Therefore, many authors use a broader definition, in which cambium comprises all dividing cells, i.e. both initials and mother cells (Wilson *et al*., 1966; Hejnowicz, 2012). However, application of this broad definition may also be problematic. For instance, as cells divide less frequently on the phloem side, one can sometimes observe undifferentiated cells resembling cambial cells in this region that do not fit into the broad definition of cambium as they do not undergo periclinal divisions. Moreover, adjoining cells belonging to different radial files may exhibit different stages of development, i.e. cells in one radial file may still divide, while cells in another file may have already started to differentiate. Figure 3 provides an example where the majority of dividing cells are located between the red dotted lines, which approximately encompass the vascular cambium according to the broad definition. Nonetheless, some cells which still undergo divisions reach far into the secondary xylem (cells marked with asterisks in Fig. 3). In this review, we therefore define cambium simply as a zone of undifferentiated cells located between the developing secondary xylem and secondary phloem; these cells do not exhibit strong growth in a radial direction (Fig. 3; zone approximated by black dotted lines). Outside the vascular cambium we can recognize developing xylem and phloem (growing and differentiating cells). Beyond this, after completion of cell development, mature secondary xylem and mature secondary phloem can be observed.

## III. MECHANICAL STRESS AND AUXIN

Auxin is a plant hormone present in the vascular cambium (Sundberg, Little & Cui, 1990) and in growing and differentiating derivative cells, where it is distributed according to a radial concentration gradient (Uggla *et al*., 1996, 1998). Auxin is key to the canalization hypothesis, which explains the basis of vascular strand formation (Sachs, 1991) and the acid growth hypothesis regarding cell wall loosening and cell elongation (Rayle & Cleland, 1992). The concentration of auxin affects the size and number of vessel elements (Aloni & Zimmermann, 1983; Zakrzewski, 1991; Aloni, 2021). It is likely that hormonal regulation and mechanical stress are linked, especially during processes involved in the growth of vessel elements and determination of the course of vessels. Nakayama *et al*. (2012) confirmed that PIN1 (PIN‐FORMED1) proteins, involved in polar auxin transport, show sensitivity to a mechanical stimulus. An increase in mechanical strain led to: (*i*) an overall increase in the amount of PIN1 transporters in a cell, and (*ii*) an increase in the fraction of transporters localized to the cell membrane (Nakayama *et al*., 2012). PIN proteins are cellular efflux transporters of auxin (Rubery & Sheldrake, 1974; Raven, 1975; Friml, 2003; Vanneste & Friml, 2009; Heisler *et al*., 2010; Nakayama *et al*., 2012). A possible mechanism by which auxin transport is linked to cell wall strain involves: (*i*) transfer of strain from the wall to the cell membrane (with extracellular receptors likely participating in this process); and (*ii*) in the areas of higher strain, auxin efflux transporters accumulate on the cell membrane, as a result of the interaction of auxin with auxin binding protein 1 (ABP1), which interferes with constitutive cycling of PINs between the plasma membrane and endosomes leading to the inhibition of clathrin‐mediated endocytosis, and possibly also as a result of increased exocytosis (Robert *et al*., 2010; Li, Friml & Grunewald, 2012; Nakayama *et al*., 2012). A recent study (Li *et al*., 2019) showed that PIN1 relocalization is initiated by mechanical stimulation which causes transient changes in cytoplasmic Ca^2+^ concentration. Therefore, it is likely that a combination of biomechanical and hormonal regulation plays a key role during the formation of vessel elements as well as whole vessels.

## IV. MECHANICAL CONDITIONS IN THE VASCULAR CAMBIUM AND DEVELOPING XYLEM

**Fig 4:**
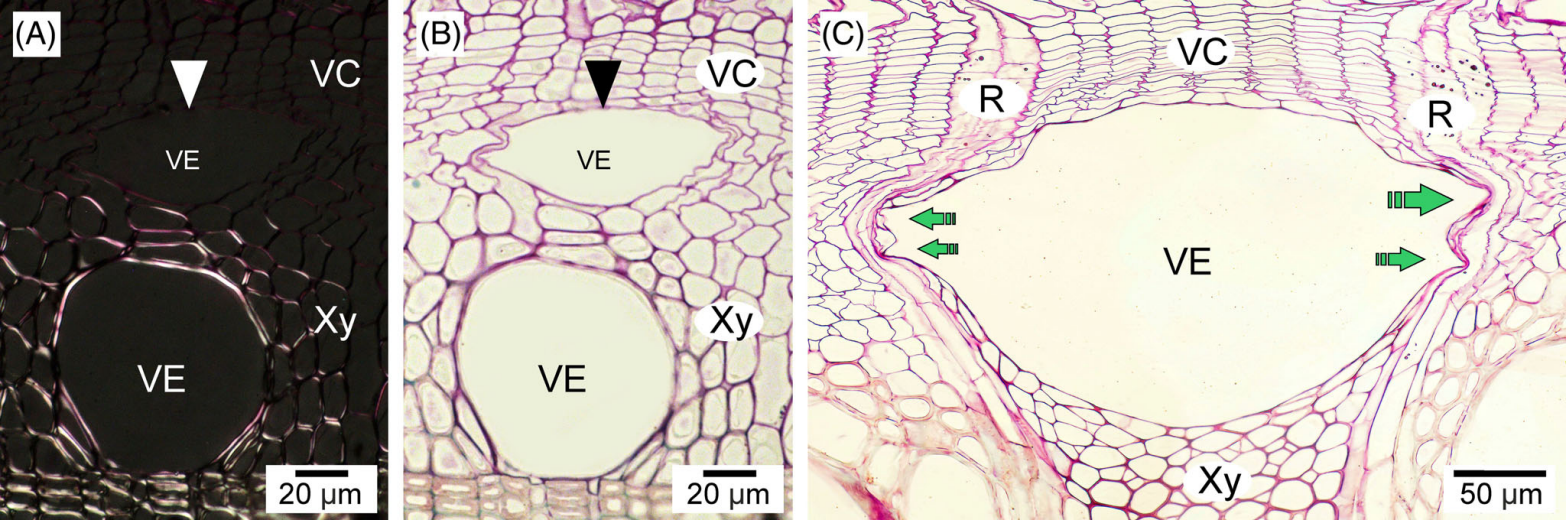
Developing broad vessel elements in ring‐porous species. (A, B) *Quercus robur* L. branch (seen under polarized light and bright‐field illumination, respectively). (C) *Robinia pseudoacacia* L. stem. Note in B that during intensive growth phases vessel elements with thin primary cell walls may exhibit a radially flattened shape. The rounded shape of broad earlywood vessel elements becomes permanent later in development. In A, the polarized light image allows visualization of the progress of maturation, i.e. deposition of secondary cell walls. White and black arrowheads in A and B indicate a fragment of primary cell wall of the developing vessel element. Green arrows in C indicate regions of strong indentations of the neighbouring rays caused by the growing vessel element. These indentations are associated with the tangential growth of vessel elements. Tangential intrusive growth of a vessel element ceases as soon as it contacts ray cells. R, ray; VC, vascular cambium; VE, vessel element; Xy, xylem.

**Fig 5:**
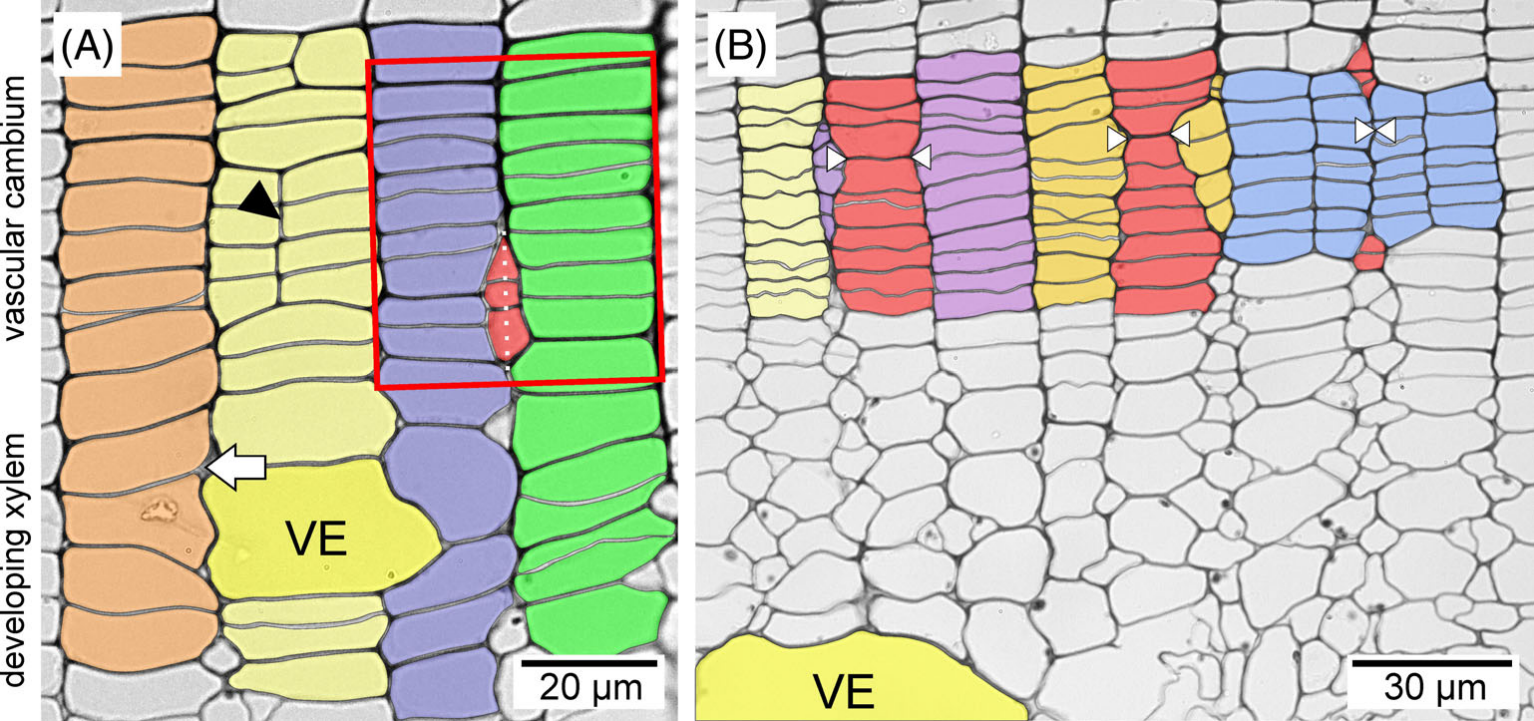
Transverse sections of vascular cambium and the zone of developing xylem of a *Robinia pseudoacacia* L. stem sample collected in July. Growth of the latewood vessel element (VE) begins near the boundary of the vascular cambium and zone of developing xylem (A) and continues in this zone (B). Note that despite rearrangement of the cambial cells in the area indicated by the red rectangle, the blue‐ and green‐coloured radial files do not move apart in the tangential direction. Therefore rearrangement of the cells did not lead to an overall increase in cambial circumference. White arrow indicates a microspace. Black arrowhead indicates an example of anticlinal division. In B white arrowheads indicate eliminations of initials in radial files (marked in red) due to intrusive growth of longitudinal edges of initials in neighbouring radial files; different stages of the elimination process are visible.

Mechanical stress is known to be extremely important for cell functioning, growth, and differentiation. For example, application of a pressure girdle to *Fagus sylvatica* stems inhibits vessel formation (Bauer & Eschrich, 1997). Zhou *et al*. (2006, 2007) observed that protoplasts placed under compressive stress elongate in the direction perpendicular to the stress, and Lintilhac & Vesecky (1984) and Lynch & Lintilhac (1997) showed that mechanical stress determines the orientation of the division plane in cell protoplasts embedded in agarose blocks. However, the patterns of mechanical stress present in the vascular cambium are still unresolved. Some authors assume that tensile stress acts in a circumferential (tangential) direction (see Fig. 1) and compressive stress acts in a radial direction (Hejnowicz, 1980, 1997, 2012; Kwiatkowska & Nakielski, 2011). According to this view radial compressive stress would result from the radial growth of xylem (growth of derivative cells following periclinal divisions in the cambium) in the presence of constraints imposed by external tissues, i.e. bark (Hejnowicz, 1980; Kwiatkowska & Nakielski, 2011). Observations of intrusively growing cells have shown that their tips are rounded (Kojs, 2013), as intrusive growth of a cell is enabled when space is created in its vicinity (Romberger, Hejnowicz & Hill, 1993; Hejnowicz, 2012), i.e. cell growth occurs into a previously created space. Vessel elements show strong intrusive growth at the border of the cambium and developing xylem (Figs 4 and 5), with this growth mainly involving their radial (anticlinal) walls (Barnett, 1992). Intrusive growth of vessel elements occurs between periclinal (tangential) walls of cells of adjacent radial files of the axial system, which can be easily deduced from the lack of continuity of these files (Wilczek *et al*., 2011*b*; Hejnowicz, 2012; Gizińska *et al*., 2021).

**Fig 6:**
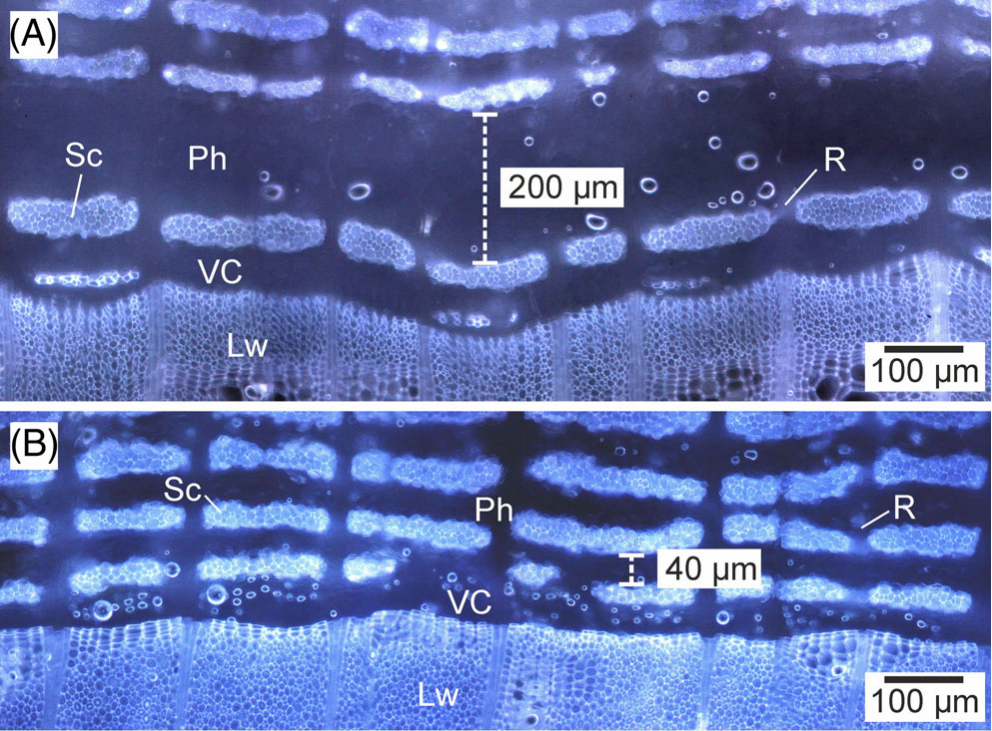
Transverse sections of *Robinia pseudoacacia* L. including secondary phloem, cambium and latewood (fluorescence technique). (A) Active stage (August). (B) Dormant stage (March). A comparison of these tissues indicates that phloem formed in one growing season collapses before growth of vessel elements begins in the following season: functional phloem between successive sclerenchyma bands has a radial dimension of approximately 200 μm in August (A), whereas the radial dimension of collapsed phloem is approximately 40 μm in March (B). Lw, latewood; Ph, phloem; R, ray; Sc, sclerenchyma fibres; VC, vascular cambium.

So how is a space into which a vessel element can grow created between the periclinal walls of cells, assuming that cambium is compressed in a radial direction (Kwiatkowska & Nakielski, 2011)? By using incision experiments, Hejnowicz (1980) identified radial tensile stress in the vascular cambium of two ring‐porous species in spring. Under such mechanical conditions, separation of the periclinal walls of cells in radial files adjacent to a growing vessel element seems possible, resulting in the formation of spaces available for intrusive growth (Hejnowicz, 2012; Kojs, 2012). Hejnowicz (1980, 1997) speculated that radial tensile stress in the cambium may result from synchronous collapse of sieve tubes, as well as companion cells, in phloem functioning in the previous growing season. The value of tensile stress acting on the middle lamellae between periclinal walls of cells would depend on the degree of synchronization of such phloem cell collapse (Hejnowicz, 1997). According to this concept radial tensile stress in the cambium would change to compressive stress later in the growing season (Hejnowicz, 1997; Kwiatkowska & Nakielski, 2011). However, the study of *Robinia pseudoacacia* xylogenesis (Miodek *et al*., 2020) revealed that the phloem within the stem seems to collapse much too early to explain the growth of first broad vessel elements of earlywood (Fig. 6) – phloem showed a reduced radial dimension in March, compared to the radial dimension observed in August of the previous year. Moreover, assuming that radial stretching of vascular cambium in ring‐porous trees is of a transient nature (occurring only in the spring) (Hejnowicz, 1997; Kwiatkowska & Nakielski, 2011): (*i*) how can one explain growth of vessel elements in latewood of ring‐porous species if the cambium is radially compressed in the later part of the growing season; and (*ii*) how can growth of vessel elements in diffuse‐porous species be explained, especially in the later part of the growing season? Hejnowicz (2012) proposed that the rate of radial growth in tissue containing a developing vessel element may differ from that of nearby vesselless regions. Temporally vesselless cambial sectors grow faster, with such growth meaning that they take over the pressure exerted by outer tissues, allowing simultaneous stretching of the sector containing the growing vessel element.

An alternative viewpoint is a hypothesis based on changes in hydration of tree tissues related to transpiration occurring during the day and its cessation at night (Kojs & Rusin, 2011; Kojs, 2012, 2013; Kojs *et al*., 2012). Numerous reports have identified diurnal cycles of plant tissue deformation – a phenomenon known as diurnal strain (e.g. Kozlowski & Winget, 1964; Simonneau *et al*., 1993; Ueda & Shibata, 2001; Yoshida *et al*., 2003) – that have been used to explain the intrusive growth of cambial cells (Włoch, Mazur & Kojs, 2001; Włoch, Mazur & Bełtowski, 2002; Kojs *et al*., 2004*a*; Kojs, Włoch & Rusin, 2004*b*; Jura *et al*., 2006). The key difference between this hypothesis and that outlined above regarding the predominance of radial compression of vascular cambium in the growing season lies in the timing and frequency of the presence of radial tensional stress within the vascular cambium. In this latter hypothesis it is assumed that there is a regular temporal pattern of radial tensile stress in the vascular cambium. In trees with developed foliage it is hypothesized that during the day radial compression of the vascular cambium would result from water loss due to ongoing transpiration. At night, or during the day in conditions that limit transpiration (e.g. cold, rain) tissues would be rehydrated and strong radial tensile stress would be created in the cambium (Kojs & Rusin, 2011; Kojs, 2012, 2013; Kojs *et al*., 2012) – xylem strain is considerably lower than phloem strain due to the higher content of cells with lignified and thick secondary walls. According to Kojs (2012), this strong radial tensile stress would allow the radial growth of cells. Therefore, radial growth might be viewed as an adaptation to alternating mechanical stress cycles (Kojs *et al*., 2012; Kojs, 2013). Furthermore, it may be speculated that prior to leaf development the cycle is reversed, i.e. tensile stress in the radial direction required for the growth of vessel elements is created during the day, and compressive stress at night, due to increased tissue hydration (i.e. changes in the osmotic potential of cells) during the day resulting from increased temperature and water availability (P. Kojs, in preparation). The experiments discussed above involving incisions of *F. excelsior* and *R. pseudoacacia* cambia (Hejnowicz, 1980) may be consistent with this hypothesis, where they were carried out during the day and before leaf development in ring‐porous species: tangential incisions of cambia were made in early May 1977 in *F. excelsior*, and in 1978 (when earlier phases of cambial reactivation were studied) in *F. excelsior* and *R. pseudoacacia* (Hejnowicz, 1980). Bud break and foliage development in ring‐porous species is often delayed in comparison to diffuse‐porous ones (Friesner, 1942; Aloni & Peterson, 1997; Panchen *et al*., 2014). Radial tensile stress in the vascular cambium associated with the diurnal cycle of tissue swelling may provide an explanation for the growth of vessel elements in both ring‐ and diffuse‐porous species during the entire growing season. Radial tension, together with shearing stress, would control the formation of microspaces between periclinal walls of cells in the cambium and at the border of the cambium and developing xylem (Kojs, 2012, 2013; see microspaces in Fig. 5).

Pectins are the main constituent of the middle lamellae. Pectins show thixotropy, meaning that they can lower their viscosity in response to mechanical force (Woodcock, 1985; Barnes, 1997) in a reversible way (Barnes, 1997). Under shearing mechanical stress pectin gels undergo elastic deformation up to a threshold value, beyond which liquid‐crystal domains are created that can flow in relation to other parts of the pectin gel (Kerst *et al*., 1990; Mujumdar, Beris & Metzner, 2002). Thus mechanical stress operating in/near the cambial tissue may lead to a reduction in viscosity of the middle lamellae, separation of parts of primary walls and thus to the formation of spaces. Mother cells of vessel elements may grow intrusively into these spaces. Kojs (2013) observed the formation of large microspaces at the border of the cambium and xylem between 3 a.m. and 5 a.m. Since sampling and anatomical investigations are not usually carried out at night, it may be that this phenomenon has been overlooked. As mentioned above, anatomical observations indicate that intrusive growth of vessel elements occurs near the border of these two tissues (Figs 4 and 5).

There is currently no consensus regarding the mechanical conditions under which cambial cells and their derivatives function and grow. Consideration of physiological aspects involving tissue swelling and shrinkage (Ueda & Shibata, 2001; Yoshida *et al*., 2003), as well as anatomical investigations concerning the precise course of intrusive growth of cambial initial cells (Włoch **&** Połap, 1994; Włoch *et al*., 2001, 2002, 2009, 2013; Kojs *et al*., 2004*a*,*b*; Jura *et al*., 2006; Karczewska *et al*., 2009; Wilczek *et al*., 2018; Miodek *et al*., 2021), have raised serious objections against earlier assumptions concerning mechanical stress in the vascular cambium.

## V. SHAPE OF ENLARGING VESSEL ELEMENTS

**Fig 7:**
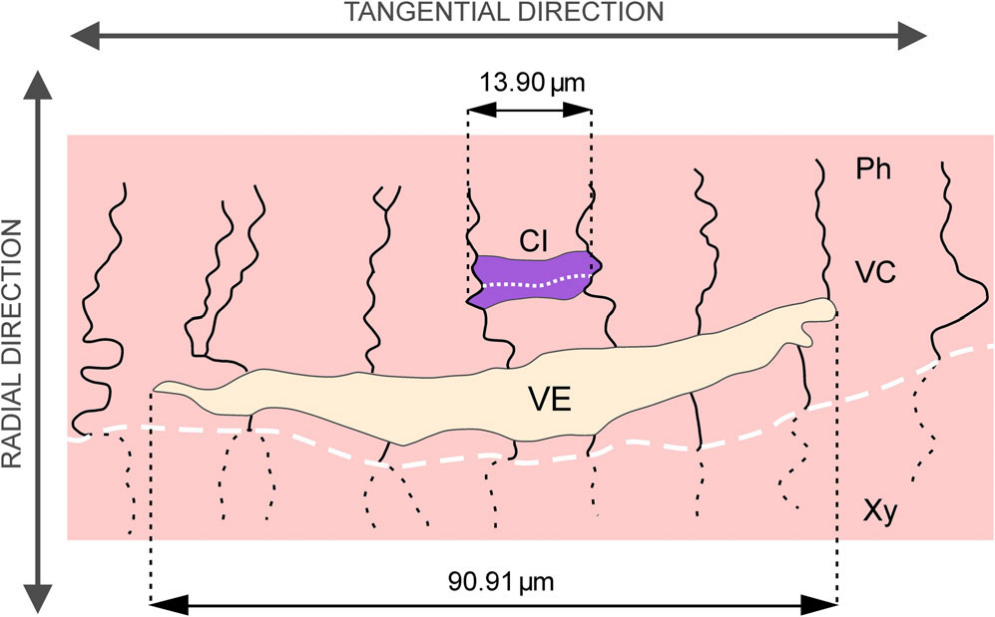
Scheme of a transverse section of a vessel element in juvenile ring‐porous wood of *Quercus robur* L. at the beginning of the growing season. The vessel element (VE) is at an early stage of development. Note that it is strongly flattened in the radial direction. Cambial cells, one of which is a fusiform initial responsible for the formation of the mother cell of the observed vessel element, are shaded in violet. These cells were distinguished on the basis of their thickness, the occurrence of periclinal division and a thin, newly formed cell wall (white dotted line). Black lines indicate anticlinal walls of cambial cells and their derivatives. Anticlinal walls of xylem cells formed in the previous season are indicated by black dashed lines. In this phase of development, the vessel element has increased its tangential dimension (compared with the tangential dimension of the cambial cells). The growth ring boundary is indicated by the white dashed line. Illustration made from a semi‐thin section of a sample embedded in epon (approximate thickness 3.75 μm). CI, cambial initial; Ph, phloem; VC, vascular cambium; VE, vessel element; Xy, xylem.

**Fig 8:**
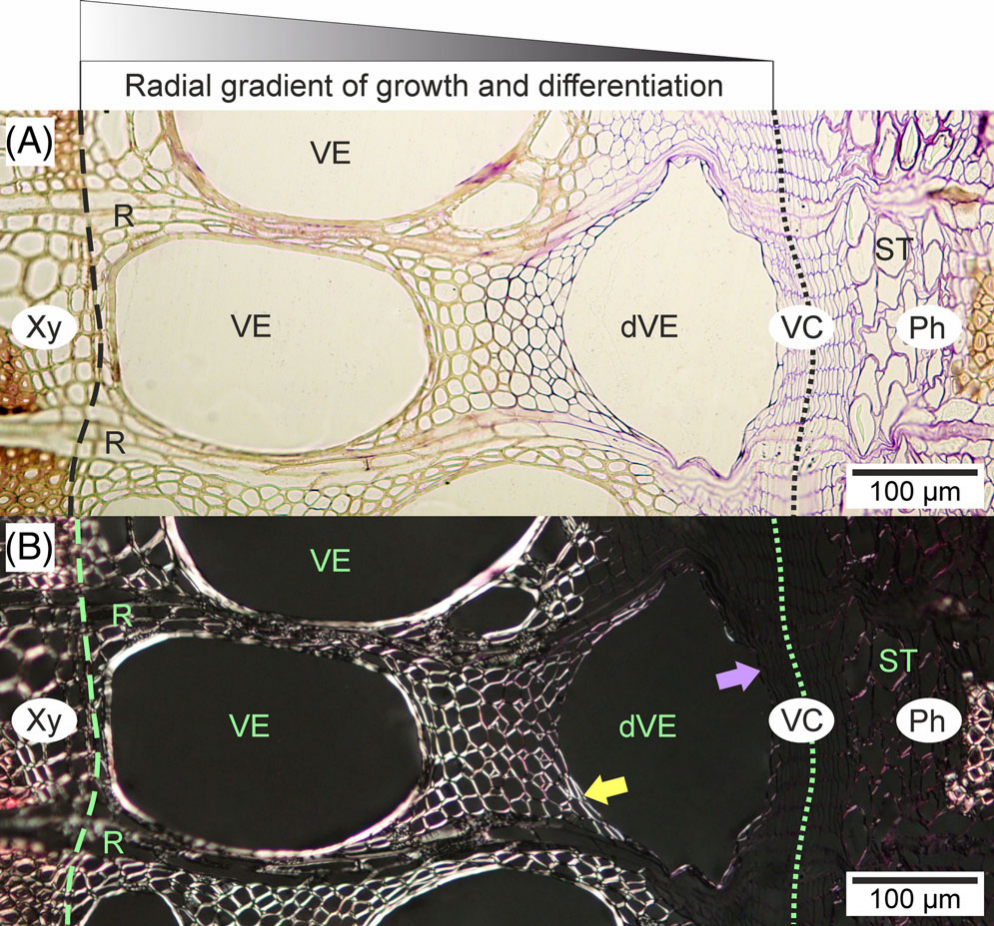
Transverse sections of vascular cambium and adjacent tissues of a *Robinia pseudoacacia* L. stem under bright field illumination (A) and polarized light (B). In B, the violet arrow indicates the primary cell wall of the developing vessel element; the yellow arrow indicates the secondary cell wall. Dashed lines indicate growth ring boundary; dotted lines show the presumed location of cambial initials. dVE, developing vessel element; Ph, phloem; R, ray; ST, sieve tube; VC, vascular cambium; VE, vessel element; Xy, xylem.

Before attaining their final transverse dimensions, broad vessel elements observed in cross section are often flattened in a radial direction (Figs 4, 5 and 7). Such vessel elements, as revealed by polarization microscopy, have a primary cell wall (or at least parts of this wall) not thickened by secondary cell wall layers (Figs 4 and 8). Initially, this flattened shape was explained as resulting from differential growth rates of vessel elements in different directions. From their examination of *Quercus rubra* tissues Zasada & Zahner (1969) stated that vessel elements first rapidly increase their tangential dimension, followed by a gradual increase in radial dimension. Hejnowicz (2012) presented a scheme showing successive stages of vessel element growth in which tangential and radial diameters become equivalent only in the final stage of vessel element development. According to Hejnowicz (2012), the occurrence of tensile stress in the radial direction would allow separation of periclinal walls of cells in radial files adjacent to a vessel element.

We suggest that in the presence of such strong radial tensile stress in the vascular cambium, the frequently observed initially flattened shape of vessel elements may not necessarily be a result of differential growth rates in the radial and tangential directions. Assuming that growth of a vessel element starts into a microspace created after radial tensile stress‐induced separation of neighbouring periclinal cell walls, and that cells grow when tissue hydration is high during the diurnal cycle (Kojs & Rusin, 2011; Kojs, 2012, 2013), it may be the case that sampling procedures affect the mechanical conditions within the tissue leading to artefacts in vessel shape. The collected tissue will no longer be subjected to strong tensile stress, some cells may lose their turgidity, and the thin primary walls of developing vessel elements could easily collapse. In Figs 4 and 8 it can be observed that broad earlywood vessel elements assume a permanent rounded, but sometimes radially elongated, shape during their later development. Figure 8 shows a radial gradient of growth and differentiation, inwardly from the vascular cambium. It is clear that the developing vessel element has a semi‐circular shape on the xylem side associated with asymmetrical secondary cell wall deposition, but an irregular shape on the cambial side (closer to the cambial initial surface) which is associated with the occurrence of a thin primary cell wall that may be prone to deformation. Additionally, in cases where the secondary walls of developing vessels are thin due to gene modification (Turner & Somerville, 1997; Hao & Mohnen, 2014), or to metabolic (Damari‐Weissler *et al*., 2009), or environmental (Aloni, 2021) stresses, collapsed and deformed vessels are formed. Note that diurnal cycles of changes in *in vivo* tissue hydration will also include periods of tissue shrinkage due to loss of water (Simonneau *et al*., 1993; Kojs & Rusin, 2011). Thus, it can be speculated that there will be periods in a diurnal cycle when mechanical conditions are not suitable for growth of vessel elements. In these periods, vessel elements with fragments of non‐lignified primary cell wall could temporarily assume a slightly flattened shape. If this flattening persists over time it may result in the formation of a peculiarly shaped (deformed) mature vessel (A. Miodek, unpublished data). Such tissue shrinkage due to loss of water (Simonneau *et al*., 1993; Kojs & Rusin, 2011) may also explain the formation of deformed/collapsed vessel elements with thin secondary cell walls. Burggraaf (1973) reported that the sampling method is critical for the observed shape of vessel elements, noting that while in earlier analyses (Burggraaf, 1972) the observed shape of vessel elements was as described by Zasada & Zahner (1969), a different sampling technique produced only circular‐shaped developing vessel elements (Burggraaf, 1973). The sampling technique proposed by Burggraaf (1973) included careful tissue collection and preparation of thick sections of developing earlywood. Thus, the techniques used for sample collection and preparation may be of great importance in the context of observations of vessel elements at early stages of their growth.

An alternative model of vessel element development was presented by Wilczek *et al*. (2011*b*), who suggested that: (*i*) vessel elements grow intrusively between periclinal cell walls; (*ii*) the whole tissue undergoes symplastic growth in a radial direction; and (*iii*) at each stage of growth, tangential and radial diameters of the growing vessel element are similar. In their model, growth of a vessel element takes place in periods when the whole cambial tissue is under radial tensile stress. If a relatively large space is created between periclinal walls as a result of strong radial tensile stress, a vessel element mother cell may start to grow to fill this space.

## VI. INTRUSIVE GROWTH OF VESSEL ELEMENTS AND CAMBIAL INITIALS

**Fig 9:**
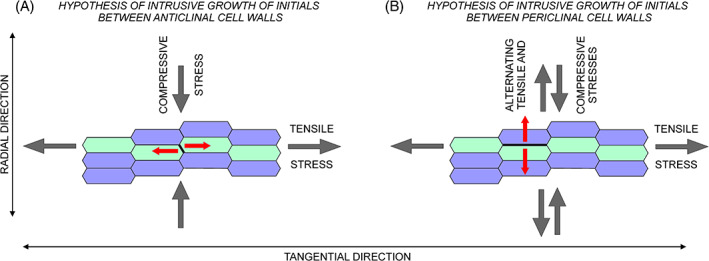
Schemes of transverse sections showing the types of stress occurring in the vascular cambium, according to two hypotheses of intrusive growth of cambial initials. These mechanical stresses may either allow the initial cell to grow intrusively between anticlinal cell walls of neighbouring initials (A) or between periclinal walls of neighbouring initials and their closest derivatives (B). (A) An initial cell located above/below the visible transverse plane would grow intrusively in an axial direction into a microspace created by separation of the anticlinal walls (marked as a thick black line). (B) An initial cell located above/below the visible transverse plane would grow intrusively in an axial direction into a microspace created by separation of the periclinal walls (marked as a thick black line). The direction in which separation occurs is indicated by red arrows. The type of stress (compressive *versus* tensile) is indicated by large, grey arrows. Cambial initials are shaded green; mother cells are blue.

Enlarging vessel elements grow intrusively in close vicinity to cambial initials. Therefore, it is of interest to consider whether cambial initials and enlarging vessel elements are subject to similar mechanical conditions and have a common mechanism of intrusive growth. There is inconsistent information available regarding the location of intrusive growth of cambial initials. Some sources state that intrusive growth of cambial initials is localized between the radial cell walls (e.g. Włoch, 1976; Evert, 2006), or imply this location by arguing that an increase in circumference of non‐storied cambium occurs through the intrusive growth of initials after oblique (pseudotransverse) anticlinal divisions (Butterfield, 1973; Zagórska‐Marek, 1981; Romberger *et al*., 1993; Evert, 2006; Hejnowicz, 2012). In order to increase the circumference of the cambial cylinder, microspaces into which cambial initial cells can grow intrusively must be created between the radial walls of cells. Thus, the cells between which an initial is growing become separated and move apart in a tangential direction (Fig. 9A). Following this concept, it was stated that cambial cells are stretched in the tangential direction (Hejnowicz, 1980, 1997, 2012; Romberger *et al*., 1993; Kwiatkowska & Nakielski, 2011). Contrary to this pattern, intrusive growth of vessel elements is thought to proceed in a different way, and under different mechanical conditions. Intrusive growth of vessel elements occurs between periclinal walls of cells as a result of radial tensile stress (Hejnowicz, 2012), while intrusive growth of initials occurs between radial walls of cells under conditions of tensile stress in the tangential direction and compressive stress in the radial direction (Hejnowicz, 1997). This disparity is confusing, since there seems to be no clear reason for these two types of cells to grow by different mechanisms.

By contrast, recent reports show that intrusive growth of cambial initials occurs between the periclinal walls of initial cells and their closest derivatives (Kojs *et al*., 2004*a*,*b*; Karczewska *et al*., 2009; Włoch *et al*., 2013; see Fig. 9B), thus in the same way as intrusive growth of enlarging vessel elements (Wilczek *et al*., 2011*b*; Gizińska *et al*., 2021). The location of intrusive growth of cambial initials between periclinal cell walls is consistent with evidence that intrusive growth does not affect the circumference of the cambial cylinder (Jura *et al*., 2006; Włoch *et al*., 2009, 2013; Wilczek *et al*., 2011*a*; Miodek *et al*., 2021; see also Fig. 5). It should be noted that growth of vessel elements is often associated with substantial changes in the arrangement of adjacent cells (Evert, 2006). As for cambial initials (Jura *et al*., 2006), the location of intrusive growth of vessel elements between periclinal walls of adjacent cells of the axial system should not increase the tangential dimension of surrounding tissue. This is logical as intrusive growth of a vessel element in the tangential direction is related to disruption of continuity of adjacent radial files (Wilczek *et al*., 2011*b*; Hejnowicz, 2012; Gizińska *et al*., 2021), or shortening of the periclinal walls of cells of the axial system (Gizińska *et al*., 2021). Interestingly, a tangential increase in diameter of a vessel element can take place not only intrusively, but also symplastically (Gizińska *et al*., 2021). This refers to specific situations, when a growing vessel element is adjacent to a ray/rays. Partial/complete separation of periclinal walls can be observed in radial files of axial system cells that are separated from a vessel element by a ray/rays (Gizińska *et al*., 2021). It can be deduced that growing vessel elements do not contribute to an increase in circumference of the developing secondary xylem.

While considering this recent evidence for the location of intrusive growth of initials between periclinal cell walls, the issue of cell elimination is pertinent. Cell elimination in the vascular cambium is now thought to occur as a consequence of intrusive growth of an initial cell between an initial depositing a radial file which will be eliminated, and its closest derivative (Kojs *et al*., 2004*a*; Jura *et al*., 2006; Włoch *et al*., 2009; Hejnowicz, 2012); elimination occurs as a result of intrusive cell growth between periclinal walls. During elimination a given initial cell loses its initial status, is removed from the initial surface and differentiates on the xylem/phloem side (Włoch & Połap, 1994; Jura *et al*., 2006; Włoch *et al*., 2013; Wilczek *et al*., 2014*a*). It is speculated that cambial initials do not actively intrude between walls of neighbouring cells (Hejnowicz, 1980, 1997), but instead grow into microspaces created between cells (Kojs, 2013; Włoch *et al*., 2013). Therefore, elimination of cells due to intrusive growth of other cells would require the occurrence of radial tensile stress, as is the case for growing vessel elements. Thus, according to recent reports, growth of both vessel elements and cambial initials as well as cell elimination in the vascular cambium are all associated with intrusive growth between periclinal walls (Jura *et al*., 2006; Karczewska *et al*., 2009; Wilczek *et al*., 2011*a*,*b*; Włoch *et al*., 2013; Gizińska *et al*., 2021; Miodek *et al*., 2021). Therefore, it seems that intrusive growth along periclinal cell walls is key to our understanding of events occurring in the cambium and its surroundings. Moreover, the mechanical conditions in which these processes take place are also similar – radial tensile stress.

The location of intrusive growth between periclinal cell walls has fundamental consequences in the context of cambial cell rearrangement in proximity to growing vessel elements. For example, intrusive growth/elimination within vesselless radial files of cells, occurring in the neighbourhood of a growing latewood vessel element may be observed in ring‐porous species (Fig. 5). It would be difficult to explain such phenomena if the vascular cambium is under constant radial compression (except for during early spring) (Hejnowicz, 1997; Kwiatkowska & Nakielski, 2011). As mentioned earlier, under the assumption that the cambium is under radial compression, it was speculated that a vessel element can grow under local radial tensile stress generated by adjacent, currently vesselless radial files (Hejnowicz, 2012). Such radial files would grow most rapidly, and thus take over the compression force exerted by the bark (Hejnowicz, 2012). However, intrusive growth/cell elimination from the initial surface can be observed in such radial rows – observations that can be easily explained if we assume that cambium is radially stretched (Kojs, 2012; Kojs *et al*., 2012), which seems to be true for ring‐porous trees in the spring (Hejnowicz, 1980). Radial stretching of the cambium may lead to the formation of microspaces (Kojs, 2012, 2013; Włoch *et al*., 2013) into which initials can subsequently grow intrusively. This would result in simultaneous elimination of initials from the initial surface (Jura *et al*., 2006), since intrusive growth of one cell and elimination of the neighbouring cell are inherently coupled and should be considered as simultaneous outcomes of cambial cell rearrangement. The cell events shown in Fig. 5 (growth of a vessel element, rearrangement of cambial cells) thus seem to be related to tensional rather than compressive radial stress.

In summary, there remains a need for a unified theoretical framework, providing a comprehensive explanation of the various processes occurring in the vascular cambium and zone of xylem/phloem cell development. There are currently different concepts on how a given cell type (e.g. wood fibre, initial cell, vessel element) grows, with some conflicting explanations partly related to the different assumptions concerning the mechanical conditions in which these cells grow.

## VII. INTRUSIVE GROWTH OF VESSEL ELEMENTS AND WOOD FIBRES

Fibres, especially those with thick secondary cell walls, are responsible for the mechanical strengthening of xylem tissue. As a result of axial intrusive growth at their tips, fibres are much longer than the fusiform initials from which they originate (Evert, 2006; Jura‐Morawiec *et al*., 2008; Hejnowicz, 2012). A recently published study concerning intrusive growth of the tips of wood fibres indicates that this takes place between the periclinal walls of adjacent cells within one or two radial rows of cells (Wilczek *et al*., 2018), in agreement with previous observations (Wenham & Cusick, 1975). Thus intrusive growth of fibres takes place between the periclinal walls as is the case for vessel elements (Wilczek *et al*., 2011*b*; Hejnowicz, 2012; Gizińska *et al*., 2021) and most likely for cambial initials (Jura *et al*., 2006; Karczewska *et al*., 2009; Włoch *et al*., 2013; Miodek *et al*., 2021).

Wilczek *et al*. (2018) also noted that wood fibres occurring near vessel elements showed limited intrusive growth, thereby confirming previous observations on the ring‐porous species *Robinia pseudoacacia* L. (Fujita, Tohyama & Harada, 1984, 1986) and the diffuse‐porous species *Acacia mangium* Willd. (Honjo, Ogata & Fujita, 2006; Yahya *et al*., 2015). Kojs (2013) suggested that the growth of wood fibres requires both radial tensile stress and shearing stresses. Radial tensile stress within the vascular cambium seems to be a necessary precondition for the growth of vessel elements to occur (Hejnowicz, 1980, 2012). Kojs (2013) assumed that the formation of large spaces by separation of the periclinal cell walls, which are immediately filled by growing vessel elements, causes local relaxation of the radial tensile stress accumulated in the surrounding tissues. This implies that the degree of relaxation of radial tensile stress might translate into the range of intrusive growth of fibres, and therefore to decreased fibre length. Wilczek *et al*. (2018) thus explained their observations by acknowledging the role of mechanical stress. In the present context it is noteworthy that in some species wood fibres do not occur, or occur infrequently, in the direct vicinity of vessel elements, since the latter are surrounded by parenchyma cells (including rays) or groups of tracheids and parenchyma cells (Metcalfe & Chalk, 1965). It may be the case that a specific type of cell into which a cambial derivative differentiates as well as its final shape and size are dependent upon the specific mechanical conditions occurring at the moment of its formation.

## VIII. FORMATION OF VESSEL ELEMENTS FROM OVERWINTERING CELLS

The existence of overwintering mother cells of vessel elements has been suggested by several studies (e.g. Barnett, 1992; Kitin *et al*., 1999; Frankenstein, Eckstein & Schmitt, 2005; Fonti, Solomonoff & García‐González, 2007). Kitin *et al*. (2003) stated that it is not possible to predict from which cambial cell a vessel element will differentiate. They also highlighted that it is not known whether positions of vessel elements are random, or whether their location is determined by a specific mechanism. However, when radial tensile stress is present, as for example in ring‐porous trees in the spring (Hejnowicz, 1980), it can be proposed that mechanical factors may play an important role in the formation of vessel elements. In this scenario radial tensile stress, together with shearing stresses, could lead to the formation of axial/tangential micro‐cracks at the border of the cambium and zone of xylem cell development (Kojs, 2013). Shearing stresses lead to increased fluidity of the middle lamellae (Leroux, 2012) between periclinal walls of cambial cells, facilitating their mechanical separation by radial tensile stress, and thereby enabling intrusive growth of vessel elements between the periclinal walls of adjacent cells (Wilczek *et al*., 2011*b*; Hejnowicz, 2012; Gizińska *et al*., 2021). Nonetheless, even if mechanical stress does cause separation of cell walls (allowing growth of selected cells), the issue of whether the future vessel element to be formed is randomly determined is still under debate.

## IX. COURSE OF VESSELS

**Fig 10:**
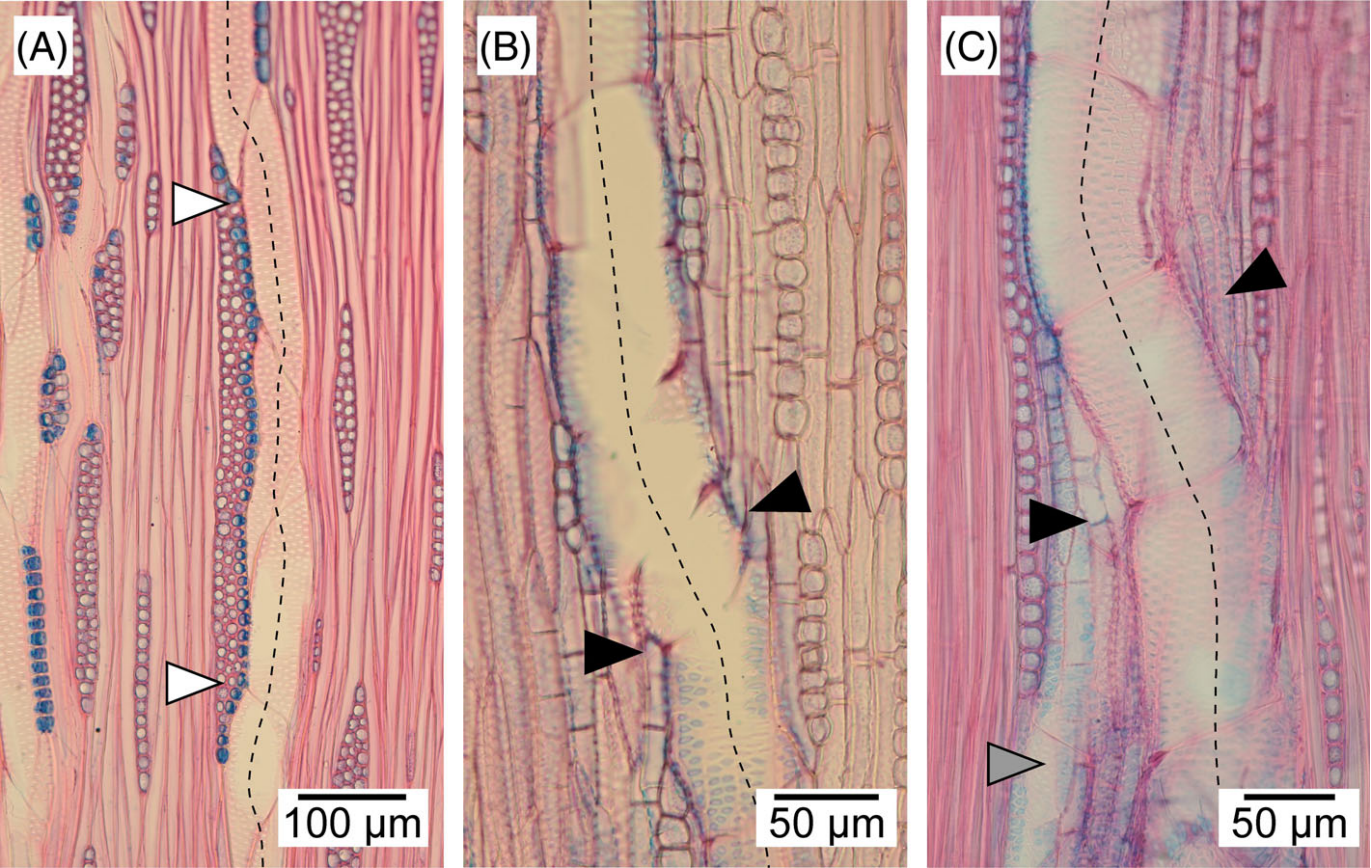
Examples of local deflections of vessels seen in tangential sections. The course of vessels is indicated by dashed lines. (A) Juvenile wood of *Acer pseudoplatanus* L. White arrowheads indicate areas where the vessel deviates slightly in the direct vicinity of a ray. (B, C) Juvenile wood of *Robinia pseudoacacia* L. Black arrowheads indicate areas where vessels change their course slightly in the absence of rays in their direct neighbourhood. In C, note the presence of a second vessel (indicated by grey arrowhead) close to the vessel identified by the dashed line.

It is widely accepted that the arrangement of the majority of xylem cells reflects the arrangement of initials in the vascular cambium at the moment of their deposition. This assumption is often made in indirect studies of cambial tissue involving analyses of xylem sections, especially for conifers which are characterized by a relatively simple wood structure with smaller variation in cell types compared to angiosperm trees (Hejnowicz & Zagórska‐Marek, 1974; Włoch, 1976; Krawczyszyn, 1977; Hejnowicz & Romberger, 1979; Wilczek, Miodek & Gizińska, 2014*b*). The relationship between the arrangement of cambial initials and vessel elements seems to be more complex, since vessel elements show intensive growth, both symplastic and intrusive, during their development (Gizińska *et al*., 2021). Moreover, vessels are interconnected to form a network, in which deflections of vessels are often observed in both a tangential and, to some extent, in a radial direction (Burggraaf, 1972; Kitin *et al*., 2004). Among factors that may affect the course of vessels, the relationship between growing vessel elements and rays is likely to be important. Vessel elements do not grow intrusively between periclinal walls of ray cells (Gizińska *et al*., 2021), and nearby rays may locally modify expansion of vessel elements (Wilczek *et al*., 2011*b*). Adjacent rays may contribute to the anisotropic growth of vessel elements and thus to local deflections of vessels in a tangential direction (Wilczek *et al*., 2011*b*; see also Fig. 10A). However, the arrangement of rays on its own is insufficient to explain all tangential deflections of a vessel. As shown in Fig. 10B, C, slight deflections in the vessel course can be observed in the absence of rays. In Fig. 10C a change in course of the vessel occurred in the vicinity of a second vessel (visible in the lower part of the micrograph). Interestingly, Kitin *et al*. (2004) pointed out that radial deflections indicate that both initiation and completion of differentiation of vessel element mother cells might not occur simultaneously. It is plausible that mechanical stress which controls the localization of PIN proteins (Heisler *et al*., 2010; Li *et al*., 2012; Nakayama *et al*., 2012; Li *et al*., 2019) may be a key factor determining the course of forming vessels. We suggest that future studies of plants should consider the mechanical stresses operating in their developing tissues.

## X. CONCLUSIONS

1. From an anatomical perspective, vessel elements grow intrusively and symplastically. Intrusive growth occurs between the periclinal walls of axial system cells, in a similar way for both wood fibres and cambial initials. It is postulated that intrusive growth of a developing vessel element between the periclinal walls of axial system cells requires the presence of radial tensile stress enabling the separation of the periclinal walls of neighbouring cells.
2. The mechanical conditions in which vessel elements grow and differentiate have not been fully elucidated. Incision experiments indicated the presence of radial tensile stress in the vascular cambium of ring‐porous species in the spring, supporting the hypothesis that radial tensile stress is present in the vascular cambium of angiosperm trees.
3. Mechanical factors seem to be vital for developmental processes occurring in the vascular cambium.
4. Clarifying the relationship between mechanical stress and auxin in the vascular cambium is particularly important for a better understanding of developmental processes in plants. Mechanical stress is a strong candidate for a key player involved not only in vessel element growth, but also in determining the course of vessels.
5. Future research should make use of methods allowing the visualization and quantification of time course of mechanical forces in three dimensions and most importantly associate them with developmental processes occurring within plants.
